# Glycoproteogenomics: A Frequent Gene Polymorphism Affects the Glycosylation Pattern of the Human Serum Fetuin/α-2-HS-Glycoprotein[Fn FN1]

**DOI:** 10.1074/mcp.RA119.001411

**Published:** 2019-05-16

**Authors:** Yu-Hsien Lin, Jing Zhu, Sander Meijer, Vojtech Franc, Albert J. R. Heck

**Affiliations:** ‡Biomolecular Mass Spectrometry and Proteomics, Bijvoet Center for Biomolecular Research and Utrecht Institute for Pharmaceutical Sciences, Utrecht University, Padualaan 8, 3584 CH Utrecht, The Netherlands; §Netherlands Proteomics Center, Padualaan 8, 3584 CH Utrecht, The Netherlands; ¶Department of Molecular and Cellular Hemostasis, Sanquin Research, Amsterdam 1066 CX, the Netherlands

**Keywords:** Glycoproteins*, Glycoproteomics, Plasma or serum analysis, Post-translational modifications*, Protein Identification*, fetuin

## Abstract

Fetuin, also known as α-2-HS-glycoprotein (gene name: AHSG), is one of the more abundant glycoproteins secreted into the bloodstream. There are two frequently occurring alleles of human AHSG, resulting in three genotypes (AHSG*1, AHSG*2, and heterozygous AHSG1/2). The backbone amino acid sequences of fetuin coded by the AHSG*1 and AHSG*2 genes differ in two amino acids including one known O-glycosylation site (aa position 256). Although fetuin levels have been extensively studied, the originating genotype is often ignored in such analysis. As fetuin has been suggested repeatedly as a potential biomarker for several disorders, the question whether the gene polymorphism affects the fetuin profile is of great interest. In this work, we describe detailed proteoform profiles of fetuin, isolated from serum of 10 healthy and 10 septic patient individuals and investigate potential glycoproteogenomics correlations, *e.g.* how gene polymorphisms affect glycosylation. We established an efficient method for fetuin purification from individuals' serum using ion-exchange chromatography. Subsequently, we performed hybrid mass spectrometric approaches integrating data from native mass spectra and peptide-centric MS analysis. Our data reveal a crucial effect of the gene polymorphism on the glycosylation pattern of fetuin. Moreover, we clearly observed increased fucosylation in the samples derived from the septic patients. Our serum proteoform analysis, targeted at one protein obtained from 20 individuals, exposes the wide variability in proteoform profiles, which should be taken into consideration when using fetuin as biomarker. Importantly, focusing on a single or few proteins, the quantitative proteoform profiles can provide, as shown here, already ample data to classify individuals by genotype and disease state.

## Abstract (precis)

Lin *et al*. applied a hybrid mass spectrometric approach to fully characterize fetuin purified from 20 individuals - 10 healthy donors and 10 septic patient donors. The results reveal that frequent genetic polymorphism influences the glycosylation pattern of fetuin. Next, the site-specific characterization of posttranslational modifications on all purified fetuins allowed to classify individuals by genotype and also disease state (i.e. sepsis). These findings provide an interesting example of glycoproteogenomics, e.g. how gene polymorphism may affect glycosylation profiles.

## Graphical Abstract

**Figure:**
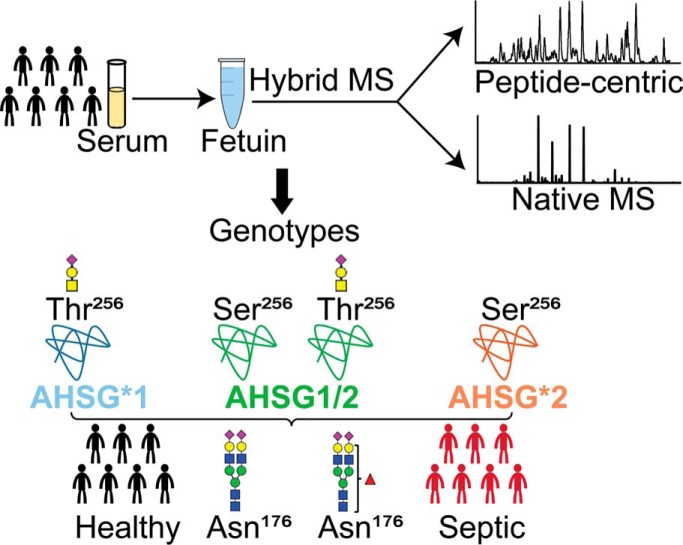

**Highlights**

- Frequent genetic polymorphism affects the glycosylation pattern of fetuin.
- Personalized in-depth proteoform profiling of fetuin purified from 20 donors.
- Classification of serum donors into three different genotypes.
- Septic patients show increased level of fucosylation at N-glycolation site N176.

Human fetuin, also known as α-2-HS glycoprotein, is an abundant glycoprotein circulating in human plasma. In adults, fetuin is secreted almost exclusively by the liver (1), but many other cells can produce this protein (2, 3). Since its discovery in 1944, human fetuin has been ascribed to various biological functions, but a concrete understanding of its exact role remains poor. One widely accepted function is its role in calcium and phosphate metabolism. Fetuin acts as one of the essential inhibitors in the prevention of ectopic mineral precipitation and deposition (4–8). Additionally, several studies have suggested a correlation between circulating fetuin levels and inflammatory, and chronic diseases, such as endotoxemia and sepsis (9), cardiovascular disease (CVD)^1^, type 2 diabetes (T2DM) (10, 11), and chronic kidney disease (CKD) (12), raising interest in its use as a protein biomarker.

Values of serum or plasma fetuin in healthy humans range from 300 to 750 μg/ml (13, 14). Fetuin concentrations seem to be independent of gender, but serum fetuin levels correlate with age and might also be influenced by genetic background (15, 16). A highly frequent genetic polymorphism of the fetuin gene (AHSG), resulting from the two common alleles AHSG*1 and AHSG*2, was already described three decades ago (17). Genomic analysis has shown that the AHSG*1 allele differs from AHSG*2 by two amino acids at positions 248 and 256, respectively. The AHSG*1 allele is characterized by ACG (Thr248) and ACC (Thr256), whereas the AHSG*2 allele is characterized by ATG (Met248) and AGC (Ser256) (18). These two abundant alleles of AHSG result in three common genotypes (AHSG*1, AHSG*2, and heterozygous AHSG1/2). The population distribution of these three genotypes varies globally. The available data from several surveys demonstrate that AHSG*1 is in frequency the foremost fetuin genotype (19–21). For example, the distribution of AHSG*1, AHSG1/2 and AHSG*2 in 697 unrelated Italians was found to be around 0.56:0.36:0.08 (22). Some studies have attempted to investigate a potential correlation between this fetuin gene polymorphism and certain diseases, however, the reported data have not been very conclusive (10, 16).

Human fetuin is a glycoprotein and its primary structure and glycosylation profile have been described (23, 24). However, the primary structure of fetuin is somehow elusive and reported discrepancies lead to confusion especially related to the absence/presence of propeptide (connecting peptide) in the matured human fetuin. According to our recent work (25), the primary structure of human fetuin contains the connecting peptide and is different from well-studied bovine fetuin. The matured human fetuin comprises an A-chain with the connecting peptide, connected to a smaller B-chain *via* a single disulfide linkage. Human fetuin is modified with a variety of posttranslational modifications (PTMs); it harbors several N- and O-glycosylations and a few phosphorylations, generating a complex mixture of proteoforms. In more detail, human fetuin harbors two N-glycosylation sites and two O-glycosylation sites in the A-chain, and one O-glycosylation site in the B-chain (25, 26). Interestingly, one A-chain O-glycan is located at position Thr256 in the AHSG*1 genotype, which is exactly the site involved in the most frequent gene polymorphism. Whether the replacement of the Thr by the Ser at position 256 in AHSG*2 affects the glycosylation pattern of fetuin has yet not been addressed. In this case, the Thr to Ser exchange still allows either amino residue to be O-glycosylated. In general, PTMs can modulate protein structure and play a key role in regulating the stability and physical-chemical properties of proteins (27, 28) and therefore there is a need to study proteoform profiles in detail. Human fetuin has been considered as a biomarker for certain metabolic diseases, but its quantitative analysis by various commercial enzyme-linked immunosorbent assays (ELISA) has been found to be problematic (29), which we hypothesize may be partly related to differences in the glycosylation patterns of AHSG*1 and AHSG*2.

Because fetuin is also known to be a negative acute phase reactant, the relationship between fetuin levels in plasma and the immune response is also of considerable interest. Glycosylation patterns of acute phase proteins may change in response to diseases such as cancer (30) and during acute inflammation. Consequently, aberrant glycosylation patterns have been related to the development or progression of disease (31–33). For example, the elevated expression of sialyl-lewis X antigen has been observed on haptoglobin, α-1-antichymotrypsin, and α-1-acid glycoprotein in response to sepsis (34, 35). Although site-specific N-glycan profiles of these acute phase proteins have been obtained before, there has been, as far as we know, no study dealing with glycosylation changes possibly occurring in fetuin during acute and chronic inflammation.

Here, we aim to describe proteoform profiles of fetuin directly isolated from serum of healthy and septic patient individuals and fill in these knowledge gaps, thereby especially focusing on glycoproteogenomics correlations, *e.g.* how gene polymorphisms may affect glycosylation profiles. This represents a challenging task, not only because of the complicated proteoform profile of fetuin, but also because fetuin has to be purified from human serum, which contains several more abundant (glyco)proteins. We demonstrated recently that using state-of-the-art hybrid mass spectrometry (MS) approaches, combining high-resolution native MS and peptide-centric MS, we may comprehensively characterize all PTMs on various therapeutic and serum glycoproteins (36–38). Making use of such methods, we previously also reported on the very distinct proteoform profiles of fetuin obtained from three different biological sources, namely plasma-derived human fetuin from a large pool of donors, recombinant human expressed in HEK-293 cells and bovine fetuin (25). Here, we first set out to extend that latter study, focusing on fetuin extracted from individuals. We therefore first purified endogenous fetuin from several individuals' serum (healthy and septic patient donors) using a one-step mixed-mode ion-exchange chromatography fractionation (IEX) (39) and performed subsequently an in-depth characterization of the proteoform profiles. We introduce an algorithm enabling semi-automated assignment of the native MS spectra based on the data gathered from the glycopeptide-centric measurements. Our data reveal, for the first time, a dramatic effect of the gene polymorphism on the glycosylation pattern of fetuin, which needs to be considered when fetuin would be used as a biomarker. Based on this knowledge we can obtain and annotate the full proteoform profiles of fetuin from 20 individuals. These proteoform profiles allow classification into the three different genotypes. Concomitantly, looking at fetuin purified from 10 healthy individual donors and 10 septic patient donors we observe significantly increased fucosylation, especially at site N176, in the samples derived from the septic patients. Our serum fetuin analysis clearly exposes the wide variability in individual proteoform profiles. However, we demonstrate that these fetuin proteoform profiles may provide data that can be used to classify individuals by genotype and disease state.

## EXPERIMENTAL PROCEDURES

### 

#### 

##### Chemicals

Dithiothreitol (DTT), iodoacetamide (IAA), trifluoroacetic acid (TFA), formic acid (FA), ammonium bicarbonate (ABC), and ammonium acetate (AMAC) were purchased from Sigma-Aldrich (Steinheim, Germany). The fetuin sample used as reference standard was purified from a pool of human serum samples and as such acquired from Sigma (α-2-HS glycoprotein; Uniprot Code: P02765, Sigma-Aldrich, Steinheim, Germany). Acetonitrile (ACN) was purchased from Biosolve (Valkenswaard, The Netherlands). Sequencing grade trypsin was obtained from Promega (Madison, WI). Gluc-C was obtained from Roche (Indianapolis, IN).

##### Individual Serum Samples

Individual human serum from 10 anonymous healthy donors was provided by Sanquin Research, Amsterdam, The Netherlands. Plasma samples were obtained after informed consent and in accordance to the ethics board of Sanquin. The whole blood from each individual was collected in a 9-ml Vacuette tube (Greiner Bio-One, Kremsmunster, Austria) containing Z Serum Clot Activator. The whole blood was then left undistributed at room temperature for 30–60 min. The clotted material was removed by centrifugation at 1800 × *g* for 20 min at room temperature and the sera were transferred as 1 ml aliquots to clean 1.5 ml Eppendorf tubes, snap froze in liquid nitrogen and stored at −80 °C. Human serum samples from 10 septic patients were acquired from Discovery Life Science, Columbus, OH, USA. All serum samples were stored at −80 °C until analysis.

##### Experimental Design and Statistical Rationale

We purified fetuin and conducted native MS and peptide-centric MS analysis of serum fetuin obtained from 20 individuals (10 healthy donors and 10 septic patient donors). Our mass spectrometric data suggested that each fetuin genotype contains unique features. These features, present in all 20 serum samples, were used for classification (using a Pearson correlation coefficient) of the donors into their genotype. Following genotypic classification, we looked at the differences in fucosylation between healthy and septic donors. For that, we applied a standard *t*-test to determine if there is a statistically significant difference between these two groups of donors.

##### Dual-column Ion Exchange Chromatography for Fetuin Purification from Individual Serum Samples

An Agilent 1290 Infinity HPLC system (Agilent Technologies, Waldbronn. Germany) consisting of a vacuum degasser, binary pump, refrigerated autosampler with 500-μl injector loop, two column compartment with thermostat, auto collection fraction module and multi-wavelength detector, was used in this study. The dual-column set-up, comprised of a tandem WAX-CAT (PolyWAX LP, 200 × 2.1 mm i.d., 5 μm, 1000 Å; PolyCAT A, 50 × 2.1 mm i.d., 5 μm, 1000 Å) two-stage column set- up. All columns were obtained from PolyLC Inc. (Columbia, USA) (39). The column compartment was cooled to 17 °C while the other compartments were chilled to 4 °C to minimize sample degradation. Mobile phase Buffer A consisted of 100 mm AMAC in water, and mobile phase Buffer B consisted of 2.5 m AMAC in water. Typically, 50 μl of serum sample was injected per run (∼3.5 mg total protein). Elution was achieved using a multi-step gradient, consisting of six transitions with increasing proportions of Buffer B: (step 1; equilibration) 0%B, 0–6 min; (step 2; salt gradient) 0–20%B, 6–11 min; (step 3; salt gradient) 20–36%B, 11–24 min; (step 4; high salt rinse) 36–100%B, 24–28 min; (step 5; high salt wash) 100%B, 28–32 min; (step 6; restoration) 100–0%B. The flow rate was set to 800 μl/min. The chromatograms were monitored by absorption at 280 nm and time-based fractions collected every 0.5 min from 19–23 min using an automated fraction collector.

##### Native MS Analysis of Fetuin

The human fetuin fraction, well separated from the most abundant serum proteins IgG and albumin, was collected from in between 22.5–23 min (see supplemental Fig. S1). This fraction, which contained about 25–30 μg of the fetuin protein, was buffer exchanged into 150 mm AMAC (pH 7.2) by ultrafiltration (vivaspin500, Sartorius Stedim Biotech, Germany) with a 10 kDa cut-off filter. The protein concentration was measured by absorbance at 280 nm and adjusted to 2–3 μm prior to native MS analysis.

Collected fetuin fraction was analyzed on a modified Exactive Plus Orbitrap instrument with extended mass range (EMR) (Thermo Fisher Scientific, Bremen) using a standard *m/z* range of 500–10,000, as described in detail previously (40, 41). The voltage offsets on the transport multipoles and ion lenses were manually tuned to achieve optimal transmission of protein ions at elevated *m/z*. Nitrogen was used in the HCD cell at a gas pressure of 6–8 × 10^−10^ bar. The MS parameters were used typically: spray voltage 1.2–1.3 V, source fragmentation 30 V, source temperature 250 °C, collision energy 30 V, and resolution (at *m/z* 200) 17,500. The mass spectrometer was calibrated using CsI clusters as described previously (40).

The accurate masses of observed proteoforms of fetuins were extracted by deconvoluting the native mass spectrum to zero-charge using the Intact Mass software (Protein Metrics ver. 1.5) (42). For the analysis of the PTMs, the data was processed manually, and glycan structures were retrieved based on known biosynthetic pathways. The average masses were used for these calculations; hexose/mannose/galactose (Hex/Man/Gal, 162.1424 Da), N-acetylhexosamine/N-acetylglucosamine (HexNAc/GlcNAc, 203.1950 Da), deoxyhexose (dHex, 146.1430 Da), N-acetylneuraminic acid (Neu5Ac, 291.2579 Da and phosphorylation (Pho 79.9799 Da). All used symbols and nomenclature are based on the recommendations of the Consortium for Functional Glycomics (43).

##### In-solution Digestion for Peptide-centric Proteomics

Around 2 μg of fetuin protein was taken from the collected fraction and dissolved into 50 mm ABC at a concentration of 1 mg/ml. The sample was then reduced with 4 mm DTT at 56 °C for 30 min and alkylated with 8 mm IAA at room temperature for 30 min in the dark. Fetuin was digested for 3 h with Glu-C at an enzyme-to-protein-ratio of 1:75 (w/w) at 37 °C and the resulted peptide mixtures were further digested by using trypsin (1:100; w/w). All proteolytic digests containing modified glycopeptides were desalted using Oasis HLB C18 cartridges, dried, and reconstituted in 20 μl of 0.1% FA before liquid chromatography (LC)-MS and MS/MS analysis.

##### LC-MS and MS/MS Analysis

All peptides (300 fmol of fetuin peptides) were separated and analyzed using an Agilent 1290 Infinity HPLC system (Agilent Technologies, Waldbronn, Germany) coupled on-line to an Orbitrap Fusion Lumos mass spectrometer (Thermo Fisher Scientific, Bremen, Germany). Reversed-phase separation was accomplished using a 100 μm inner diameter 2 cm trap column (in-house packed with ReproSil-Pur C18-AQ, 3 μm) (Dr. Maisch GmbH, Ammerbuch-Entringen, Germany) coupled to a 50 μm inner diameter 50 cm analytical column (in-house packed with Poroshell 120 EC-C18, 2.7 μm) (Agilent Technologies, Amstelveen, The Netherlands). Mobile-phase solvent A consisted of 0.1% formic acid in water, and mobile-phase solvent B consisted of 0.1% formic acid in ACN. The flow rate was set to 300 nL/min. A 45 min gradient was used as follows: 0–5 min, 100% solvent A; 13–44% solvent B within 20 min; 44–100% solvent B within 3 min; 100% solvent B for 1 min; 100% solvent A for 17 min. For the MS scan, the mass range was set from 375–1500 *m/z* at a resolution of 120,000 and the AGC target was to 4 × 10^5^. For the MS/MS measurements, electron-transfer combined with higher-energy collision dissociation (EThcD) (44) was used and performed with a normalized collision energy of 35%. For the MS/MS scan, the mass range was set from 125–2000 *m/z*; the AGC target was set to 5 × 10^4^. The precursor isolation width was 1.6 Da, and the maximum injection time was set to 200 ms.

##### LC-MS and MS/MS Data Analysis

The raw data files containing MS/MS spectra of fetuin peptides were processed using Byonic software (ver 3.2.0) (Protein Metrics Inc.) (45). The following parameters were used for data searches in Byonic: precursor ion mass tolerance, 10 ppm; product ion mass tolerance, 20 ppm; fixed modification, Cys carbamidomethyl; variable modification: Met oxidation, Ser, Thr and Tyr phosphorylation, and both N- and O-glycosylation from mammalian glycan databases; the allowed number of miscleavages was set to 4. Trypsin (C-terminal RK) and Glu-C (C-terminal DE) enzyme specificity search was chosen for all samples. The fasta file used for the peptide searches contained both the AHSG*1 and AHSG*2 fetuin amino acid sequences (UniProtKB - P02765, FETUA_HUMAN, AHSG*1 has Thr-248/Thr-256; AHSG*2 has Met-248/Ser-256). Byonic peptide cut-off score of 200 was used and all PTM-modified identified spectra were further manually inspected. Site-specific quantification of the fetuin PTMs was performed as follows; the first three isotopes were taken from each manually validated peptide proteoform for the calculation of the peak areas. Each peptide that contained modified sites was normalized individually so that the sum of all its proteoform areas was set to 100%. The average peptide ratios from all measurements were taken as a final estimation of the abundance. The XICs were obtained using the software Skyline (ver 4.2.0.18305) (46). The glycan structures of each glycoform were manually annotated. Hereby, reported glycan structures are depicted without the linkage type of the glycan units because our acquired MS/MS data do not directly provide such information.

##### Automated Site-specific Proteoform Annotation in Native MS Spectra Using Peptide-centric Data

We annotated in a site-specific manner each proteoform represented by peaks in the native MS spectra using the relative abundance of all PTMs sites derived from the peptide-centric data (the algorithm is made publicly available and supplemented at https://github.com/juer120/NativePTMannotation). The process of annotation involved two major steps: (1) the relative abundances of all possible PTM combinations on the proteoforms are retrieved from the peptide-centric data; (2) the masses of all proteoforms are extracted from the native MS spectra and then matched with the calculated proteoforms consisting of the PTM combination with the highest relative abundances, and mass window centered around detected proteoform within 100 ppm.

In detail, we first input the mass of the protein backbone retrieved from the protein sequence corrected for the presence of disulfide bridges (F_pb_) and then calculate the theoretical *m/z* of all proteoforms in determined charge state (z) by permuting masses of all PTMs identified from peptide-centric measurements. The *m/z* of a proteoform F^z+^ can be calculated as:

$$F^{z+}=\left(\text{Fpb}+\sum_{i=1}^{n}fij+z\times 1,007276\right)/z$$

where n is the number of PTM sites; f_ij_ is the mass of the j-th modification at site i. Second, we calculate the relative abundance P of a proteoform F^z+^ with all possible PTM combinations, which is calculated by:

$$P=\prod_{i=1}^{n}Pij$$

where Pij is the normalized relative abundance of the j-th modification at site i; n is the number of PTM sites. Pij is calculated by:

$$Pij=\frac{Vij}{\sum_{j=1}^{k}Vij}$$

where k is the number of possible PTM isoforms at site i (including unoccupied site); Vij is the abundance of j-th modification at site i, which is calculated based on the XIC. Third, we input all *m/z* of proteoforms (M^z+^) extracted from raw native MS spectrum and then match them with theoretical *m/z* (F^z+^) within 100 ppm, producing a list of all matched *m/z*. Lastly, we export the highest relative abundance of PTMs combination corresponding to the matched *m/z* in the list.

## RESULTS

### 

#### 

##### Native Mass Spectra and Peptide-centric Analysis of Fetuin Purified from Healthy Individuals

**Fig. 1.**
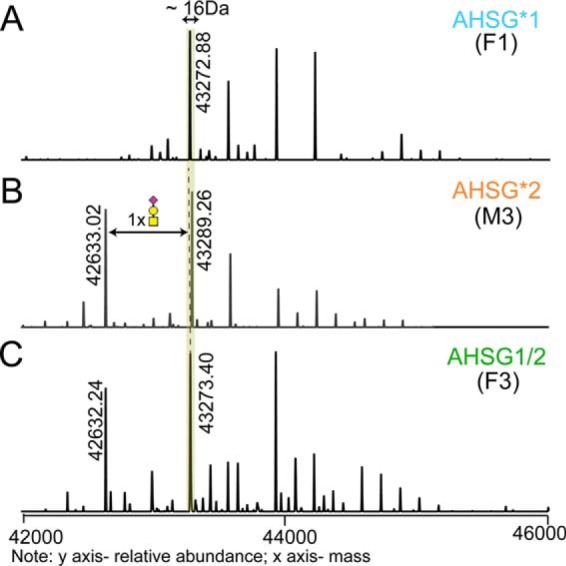
**Different genotypes lead to different proteoform profiles.** Three representative zero-charge deconvoluted native mass spectra of fetuin purified from serum of *A*, donor F1 (AHSG*1), *B*, donor M3 (AHSG*2), and *C*, donor F3 (AHSG1/2). Corresponding proteoforms between AHSG*1 and AHSG*2 differ in mass from each other by 16 Da (as shown for the proteoforms with the mass of 43,272.88 Da in *A* and 43,289.26 Da in *B*). In *B*, the proteoform with the mass of 42,633.02 Da differs from the most abundant proteoform by 656 Da, which agrees to an O-glycan composition of HexNAc_1_Hex_1_Neu5Ac_1_. This proteoform (42,632.24 Da) can be observed in *C*, but is fully absent in *A*.

**Fig. 2.**
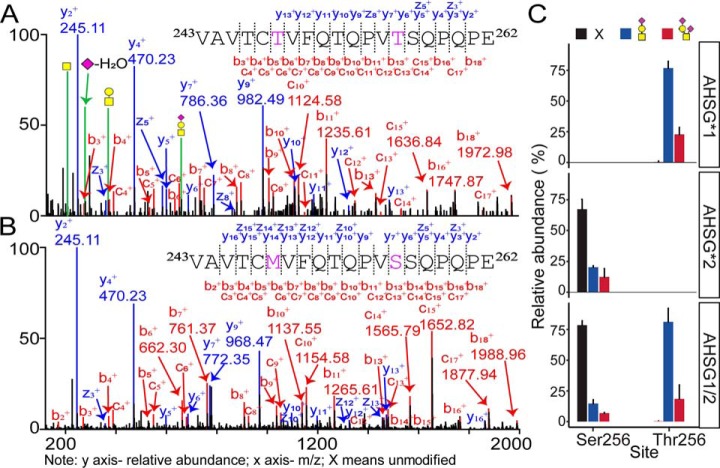
**Peptide signatures of distinct fetuin genotypes.** EThcD MS/MS spectra of the peptide *A*, ^243^VAVTC**T**VFQTQPV**T**SQPQPE^262^ (AHSG*1) and *B*, ^243^VAVTC**M**VFQTQPV**S**SQPQPE^262^ (AHSG*2), obtained by proteolytic digestion (Trypsin + Glu-C), harboring the two mutation sites. The EThcD spectra provide confirmation of the unique peptide sequence and position of the O-glycan, being Thr256 in AHSG*1. Spectra were acquired for the precursor ions with three charges and *m/z* of 958.78, and 745.03 for AHSG*1 and AHSG*2, respectively as these peptides were predominantly non-modified for AHSG*2 and fully modified for AHSG*1. *C*, Quantification of the peptide signatures, containing the mutations and O-glycosylation site Thr/Ser256. In these bars, the abundance of these peptides was averaged over all genotype-specific fetuin samples purified from the serum of the 10 healthy individuals across all three genotypes, AHSG*1 (*n* = 4), AHSG*2 (*n* = 2) and AHSG1/2 (*n* = 4). Relative abundances of peptide proteoforms were estimated from their corresponding extracted ion chromatograms (XICs) and normalized to 100%. The AHSG*1 and AHSG*2 signature peptides contain the Thr256 and Ser256 site, respectively. Both peptides can be extracted and separately quantified for the heterozygote AHSG1/2 donors. Black bar is unmodified, blue bar is modified by HexNAc_1_Hex_1_ with one sialic acid and red bar is modified by HexNAc_1_Hex_1_ harboring two sialic acids.

We initiated our study characterizing in detail human serum fetuin proteoform profiles of 10 randomly selected healthy individuals (Table I). We soon found out that these individuals harbor different fetuin genotypes and started to investigate the effect of this genetic polymorphism on the fetuin glycosylation pattern. Because fetuin is produced by at least two frequently occurring variants of the gene AHSG, AHSG*1, and AHSG*2, one could expect to observe distinct proteoform profiles of fetuin isolated from these individuals. To investigate this, we first loaded 50 μl of human serum of each individual on the dual mixed-mode column ion exchange chromatography system, comprising a weak cation exchange (CAT) column preceded by a weak anion exchange (WAX) column. Using this chromatographic set-up for serum, we collected in each run a unique fraction containing fetuin, which was subsequently concentrated, buffer exchanged, and subjected to high-resolution native MS (supplemental Fig. S2). Fig. 1 displays three representative zero-charge deconvoluted native mass spectra originating from individuals with the three different genotypes of fetuin (donor F1 - AHSG*1, donor M3 - AHSG*2, and donor F3 - AHSG*1/2). The grouping of all acquired spectra was done based on signature peaks specific for each genotype, as explained in more detail below. Although all three native mass spectra appeared to be quite distinct, many of the peaks were present in each spectrum and differed from each other primarily in signal intensity. Nevertheless, a closer look into the spectra revealed some key differences. One intense peak with the mass of 42,633.02 Da detected in AHSG*2 and AHSG*1/2 (42,632.24 Da) was completely missing in all AHSG*1 (Fig. 1*A*, and supplemental Fig. S2*A*). Therefore, this peak was specific for AHSG*2 and AHSG*1/2, and its complete absence defined unambiguously AHSG*1. Interestingly, the missing proteoform in AHSG*1 seemed to represent a significant difference in the proteoform profile between AHSG*1 and AHSG*2 and hinted at a substantial influence of the genetic polymorphism on the glycosylation profile. Additionally, when comparing corresponding peaks between AHSG*1 and AHSG*2, the mass difference of 16 Da could be observed (Fig. 1*A* and 1*B*, supplemental Fig. S2*A* and S2*B*). This is because of the mass difference between the backbone amino acid sequences of AHSG*1 and AHSG*2, exactly 15.99 Da. The native mass spectra of fetuin from the heterozygote genotype AHSG*1/2 donors showed more complex proteoform profiles (Fig. 1*C*, supplemental Fig. S2*C*) compared with the other two genotypes. This is expected, as it should be a combination of all the proteoform profiles observed for AHSG*1 and AHSG*2. In theory, we should detect double peaks for all proteoforms that are common for AHSG*1 and AHSG*2. Nevertheless, because of the very small *m/z* difference between both AHSG genes (16 Da, *i.e.* 1.23 Th for the 13^+^ charge state), we observed in practice broader or partially split peaks in the native mass spectra obtained for the heterozygote donors. Resolving such a small mass difference would require ∼10 times higher mass resolving power in the mass region around 4000 *m/z*. Our findings from the native mass analysis could be further supported and explained with the subsequent (glyco)peptide-centric analysis of all individual donors' fetuins. We digested fetuins from all three genotypes with trypsin and Gluc-C, which resulted in a set of peptides for subsequent sequencing by LC-MS/MS analysis. Data interpretation provided information about the site location, PTM type, composition and abundance of all identified peptides (for manually annotated MS/MS spectra see supplemental File S1 and S2; for Byonic searches and MS/MS spectra see Data Availability). Fig. 2*A*, 2*B* and supplemental File S1 displays annotated EThcD MS/MS spectra of two peptides with amino acid sequence ^243^VAVTCTVFQTQPVTSQPQPE^262^ and ^243^VAVTCMVFQTQPVSSQPQPE^262^, respectively signature peptides derived from proteolytic digestion of AHSG*1 and AHSG*2. These peptides contain the two aforementioned genotypic sites. Notably, manual inspection of the XICs obtained from all three fetuin genotypes revealed that Thr256 on the AHSG*1 peptide was fully occupied with O-glycans containing 0, 1 or 2 sialic acids connected to the core structure HexNAc_1_Hex_1_, whereas Ser256 on the AHSG*2 peptide was found mostly to be non-modified (Fig. 2*C*, supplemental Fig. S3). Thus, although Ser256 was marginally modified by O-glycan, it differs significantly from Thr256 which was found to be fully O-glycosylated. The relative abundance of all detected variants of the peptides containing these sites across the three different genotypes is displayed in Fig. 2*C*. The relative quantification of the site occupancy of Thr/Ser256, as obtained for all 10 studied healthy individuals, was processed using Skyline and provided in supplemental Table S1. From the peptide-centric data, we could conclusively explain the origin of the mass difference between the peaks 43,272.88 Da in AHSG*1 and 42,633.02 Da in AHSG*2 (HexNAc_1_Hex_1_Neu5Ac_1_ - 15.99).

**Table I:** Overview of donor characteristics. The first column lists the used sample names

Sample name	Sex	Age	Gene type	Sample type
M1	M	48	AHSG*1	Healthy
F1	F	37	AHSG*1	Healthy
F2	F	26	AHSG*1	Healthy
F5	F	63	AHSG*1	Healthy
M2	M	55	AHSG*2	Healthy
M3	M	54	AHSG*2	Healthy
F3	F	33	AHSG1/2	Healthy
F4	F	27	AHSG1/2	Healthy
M4	M	27	AHSG1/2	Healthy
M5	M	63	AHSG1/2	Healthy
F6	F	68	AHSG*1	Sepsis
F7	F	19	AHSG*1	Sepsis
F8	F	73	AHSG*1	Sepsis
M7	M	18	AHSG*1	Sepsis
F9	F	33	AHSG*1	Sepsis
F10	F	82	AHSG*1	Sepsis
M8	M	47	AHSG1/2	Sepsis
F11	F	55	AHSG1/2	Sepsis
F12	F	33	AHSG1/2	Sepsis
F13	F	37	AHSG1/2	Sepsis

##### Site-specific PTMs Annotation of the Native Mass Spectra

**Fig. 3.**
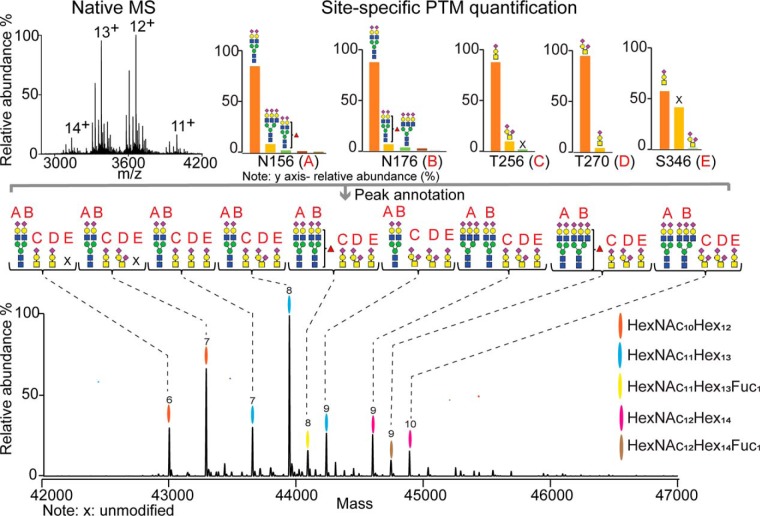
**Comprehensive, quantitative and site-specific annotation of fetuin proteoform profiles.** The site-specifically annotated zero-charge deconvoluted native mass spectrum of fetuin pooled from human sera of various donors. The overall PTM compositions of the most abundant proteoforms are color coded. Each color represents a glycan composition without the sialic acids; the number of sialic acids attached is marked on the top of each peak. All displayed proteoforms contain one phosphate moiety. The site-specific annotation of the 5 glycosylation sites present on fetuin (A = Asn156, B = Asn176, C = Thr256, D = Thr270, and E = Ser346) was assigned by using our in-house developed software, making use of the integration of the native MS and peptide-centric MS data. The complete list with all annotated proteoforms and their relative abundance can be found supplemental Table S2.

We introduced earlier an algorithm facilitating the integration of the data from native mass spectrometry and peptide-centric LC-MS/MS data, which allowed us to assess the integrity of the glycopeptide characterization though *in silico* construction of an intact proteoform profile from all combined peptide-centric data (36). The algorithm lists all possible combinations of proteoform masses based on observed PTMs, at the peptide-centric level, and eventually constructs simulated native-like mass spectra, which can be correlated to the experimental native mass spectra. However, in the earlier approach the resulted list containing all predicted proteoforms, with the total PTM composition, did not contain any site-specific information on the PTMs. Here, we further extended our algorithm (made publicly available) and applied it for the semi-automated annotation of PTMs to all the peaks in the native mass spectra, now also including site location. To perform this more advanced assignment, we amended our in-house scripts written in R (see Methods section for the detailed description). In short, we first generated a library of proteoforms with specific masses and probability ranks using the data (masses and relative intensities) derived from the peptide-centric analysis. All proteoform mass combinations were ranked according to the intensity of the detected PTM peptides from which they were constructed and the match between their theoretical and experimental masses. This means that proteoforms with the same masses (within 100 ppm), but different peptide combinations, were sorted based on the better mass match and intensity of the peptides (meaning that closer mass matches and higher intensities do get higher ranks). Next, we discarded all peaks with a relative intensity of less than 5% (related to the base peak) in the native mass spectra. The masses of peaks were then matched to the generated library with the highest ranked proteoforms. This allowed us to visualize the most probable proteoforms in the native mass spectra with their PTM composition in a site-specific manner. Fig. 3 shows such a visualization for fetuin which was purified from a pool of donors of all different genotypes (our reference sample). Cumulatively, based on our experimental data, we defined on fetuin one fixed modification (Ser138 phosphorylation) and five other PTM sites (A = Asn156, B = Asn176, C = Thr/Ser256, D = Thr270, and E = Ser346). The script operated with the fetuin backbone mass corrected for the mass shift induced by the disulfide bonds (−12 × 1.0079 Da) and one phosphate (+79.97) moiety (Ser138). Peaks in the native mass spectra were then automatically annotated based on the library containing the PTM combinations with the highest rank. The result contained a list of proteoforms with their masses, the most probable PTM combinations and site-specific information as shown in supplemental Table S2.

##### Site-specific Annotation Fully Explains Differences Observed Among AHSG*1, AHSG*2 and AHSG*1/2 Fetuin

The native mass spectra obtained from genotype-representative individuals with site-specifically annotated proteoforms defining each genotype (*i.e.* F1/AHSG*1, M3/AHSG*2, and F3/AHSG*1/2) are provided in supplemental Fig. S4. Some major differences observed across all individuals defining fetuin genotypes were already indicated above. However, the exact differences among them could be only clarified after site-specific characterization of all proteoforms. The most striking one represented the high-intensity peak with a mass of 42,633.02 Da, which is completely missing in fetuin from AHSG*1 donors and its composition consisted of two biantennary N-glycans in positions A and B (2 x HexNAc_4_Hex_5_Neu5Ac_2_) and one O-glycan in position D (HexNAc_1_Hex_1_Neu5Ac_2_). We explained the absence of this proteoform in AHSG*1 above, being due to the complete occupation of position C (Thr256) O-glycan-site. This was in pronounced contrast with position C (Ser256) in AHSG*2, which was found to be occupied only partially. The second major difference between AHSG*1 and AHSG*2 was defined by their different protein backbone mass induced by the two mutations, resulting in the exact mass difference of 15.99 Da (37,177.01–37,193.00). This could be extracted already from the two most intensive proteoforms in the native mass spectra of F1 (AHSG*1) and M3 (AHSG*2) displayed in Supplemental Fig. S4*AB*. Although both proteoforms had the same total PTM composition, site-specific annotation also revealed a difference in the O-glycan positions occupancy. Donor F1 had O-glycans at positions C and D, whereas M3 at positions D and E. A complete list of site-specifically annotated proteoforms from the three individuals representing different genotypes can be found in supplemental Tables S3–S5. In F1, we could annotate 16 proteoforms (supplemental Table S3), in M3, 15 proteoforms (supplemental Table S4), and lastly in the heterozygote donor F3 34 proteoforms (supplemental Table S5). The proteoform profiles differed from each other not only by their complexity, but also by the relative abundances of peaks.

**Fig. 4.**
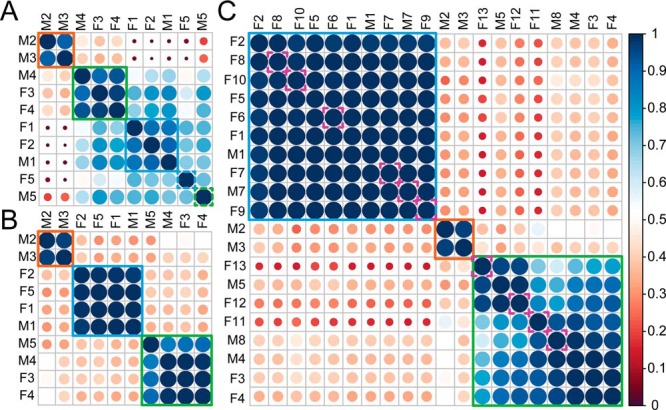
**Classification of fetuin proteoform profiles by hierarchical clustering.** *A*, Unsupervised clustering of fetuin proteoform profiles derived from the 10 healthy individuals based on the correlation between their native mass spectra. *B*, Clustering of fetuin proteoform profiles derived from 10 healthy individuals based on specific signature peaks (see supplemental Fig. S4). Color and size of the circles represent the similarity between the proteoform profiles of different genotypes. Orange, blue and green boxes represent fetuin originating from donors representing the AHSG*2, AHSG*1 and AHSG1/2 genotypes, respectively. Comparing the results in *A* and *B* the classification in *A* is distorted by two outliers derived from donor F5 (AHSG*1) and M5 (AHSG*2), respectively caused by a major change in fetuin phosphorylation in these two individuals. *C*, Clustering of fetuin proteoform profiles derived from 10 healthy and 10 septic patients based on specific signature peaks, where pink dashed line boxes indicate fetuin derived from septic individuals.

Having annotated the three representative native mass spectra, we attempted a classification of all 10 studied healthy individuals based on their proteoform profiles, following a similar approach as used earlier to classify glycol-engineered erythropoietin variants (47). We initially used unsupervised hierarchical clustering to construct a matrix based on all native mass spectra, as shown in Fig. 4*A*. This already resulted in three distinctive clusters, but also two clear outliers (donors F5 and M5). The first cluster represented fetuins from AHSG*2 donors (orange box). The second (green box) and third cluster (blue box) contain fetuin from AHSG1/2 and AHSG*1 donors, respectively. Upon closer inspection, the native mass spectra of the two outlier samples (F5 and M5), displayed more complex proteoform profiles, with nearly all peaks coexisting in pairs differing from each other by 80 Da (supplemental Fig. S2). From the LC-MS/MS data we could conclude that these two donors have a high occupancy of a second phosphorylation site, namely Ser330 (supplemental File S2). The presence of this extra phosphate moiety interfered with the genotype classification based on the complete proteoform profiles. Nevertheless, as we above identified characteristic proteoforms defining each genotype, we repeated the classification, but now using only these signature peaks. The result is shown in Fig. 4*B*, where the orange, green and purple boxes represent the genetic variants of fetuin. This data clearly exemplifies that, over a sample size of 10 healthy individuals, distinctive glycosylation patterns can be linked to the distinctive fetuin genotypes.

##### Proteoform Profiles of Fetuin from Septic Patients Donors

**Fig. 5.**
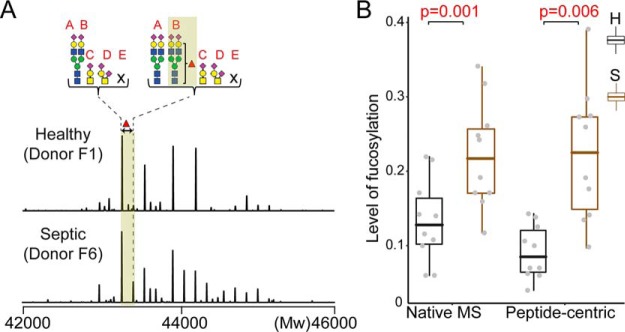
**Septic patients display enhanced fucosylation on Asn176.** *A*, Two representative zero-charge deconvoluted native mass spectra of a healthy donor (F1) and septic patient (F6). The average intensities of the site-specifically assigned fucosylated proteoform and its non-fucosylated variant were used for the determination of the relative fucosylation level. The intensities were extracted from the native mass spectra using the corresponding ion signals detected in the 12^+^ and 13^+^ charge states. *B*, Comparison of the extent of fucosylation obtained from the native mass spectra and peptide-centric analysis. The level of fucosylation in the peptide-centric data was determined by ratios of the peak areas of the fucosylated and non-fucosylated peptide containing the sepsis affected N-glycosylation site Asn176. Both approaches resulted in a statistically significant separation of the healthy and septic patients with *p* values of 0.001 and 0.006, respectively. (A = Asn156, B = Asn176, C = Thr256, D = Thr270, and E = Ser346).

Alternation of glycosylation can be a trademark of inflammation, as reported to occur on several acute phase proteins; α-1-acid glycoprotein, haptoglobin, and α-1-antichymotrypsin (34, 35). However, such data for fetuin is sporadic (48). To investigate, whether fetuins' glycosylation pattern also alters upon acute inflammation, we next purified serum fetuin from 10 individual septic patient donors (Table I) and conducted the same mass spectrometric analysis as before. Our classification strategy quickly classified all 20 native mass spectra (from the 10 healthy and 10 septic patient donors) into the three genotypes (Fig. 4*C*). This classification could be further corroborated by the peptide-centric LC-MS/MS data (supplemental Table S6). Next, we focused on whether we could classify the healthy and septic patient donors. Fetuin obtained from septic patient donors contained some molecular species with a prominently increased intensity originating from proteoforms with fucosylated N-glycans. Zooming in to this, we focused on a specific fetuin fucosylated proteoform and its non-fucosylated variant and used the ratio of their peak intensities to estimate the level of fucosylation (Fig. 5*A*). We calculated from the native mass spectra the average intensities of the two proteoforms using their peaks detected in the 12^+^ and 13^+^ charge states (supplemental Table S7). The peptide-centric LC-MS/MS analysis allowed us to pinpoint the by sepsis most affected N-glycosylation site as Asn176 (B). The ratios of peak areas of the fucosylated to nonfucosylated peptide containing this site validated the clear trend of fucosylation increase in septic patients (Fig. 5*B* and supplemental Table S8). This change in proteoform profile can thus be used to classify septic patients from healthy donors with good statistical values (*p* < 0.006).

## DISCUSSION

Here we combined high-resolution native mass spectrometry with peptide-centric (glyco)proteomics to analyze the medium abundant serum glycoprotein fetuin, which we were able to directly purify from serum of 20 donors, 10 healthy controls and 10 septic patients. From the data, it became apparent that the samples were derived from people with distinct frequently occurring genotypes, that all result in a different set of fetuin proteoforms. We further investigated in detail the variations in proteoform profiles caused by the donors' specific genotypes, providing an interesting example of glycoproteogenomics. We next also extended our analysis to serum fetuins purified from septic patient donors.

### 

#### 

##### Replacement of Thr in AHSG*1 to Ser in AHSG*2 Changes O-glycosylation Occupancy

In both the native MS and LC-MS/MS data we observed that the mutation in position 256, which is a Thr in AHSG*1 and a Ser in AHSG*2, had a dramatic effect on fetuin O-glycosylation, primarily caused by the diminished occupancy of O-glycans on AHSG*2/Ser256. Notably, both a Thr and Ser can be modified by O-glycosylation, but our finding that the O-glycosylation is reduced on fetuin Ser256 compared with Thr256 is in line with a meta-analysis on O-glycosylation occupancy, which reported a considerable preference for Thr over Ser (49). We provide here the first experimental evidence showing how the replacement of Thr for Ser in fetuin changes the occupancy of this site. In general, changes in O-glycosylation may affect protein stability or protection against proteolytic activity (50). Impact of gene mutation, including SNPs, on protein glycosylation has been reported earlier, albeit only in a few studies (51–53). Notably, such mutations have been associated with altered physiological states, especially the ones leading to disease. Whether different the here reported proteoform profiles of fetuin have biological implications needs to be further investigated.

##### Coupling Between Fetuin Backbone Processing and Phosphorylation

The native mass spectra of two healthy donors, F5 and M5, attracted our attention because of their quite distinctive proteoform profiles, representing the two outliers in Fig. 4*A*. We observed that this was primarily caused by the site-occupancy of the additional fetuin phosphorylation site Ser330. Human serum fetuin is predominantly present as singly phosphorylated, with a site-occupancy of nearly 100% at Ser138 (25). Earlier phosphoproteomics analysis has revealed that at least four more Ser residues can be partially phosphorylated (54–56). In a recent study comparing obese versus lean individuals, phosphorylation on fetuin Ser330 was attributed with a metabolically active pool of human fetuin, within the larger total pool of circulating fetuin (57). Interestingly, this Ser330 phosphorylation site is in the 40-amino acid residue long propeptide (also termed “connecting peptide”), which is believed to be lost during posttranslational processing in serum. Data from literature has suggested that fetuin can indeed exist in human serum in two processed forms, with- or without the connecting peptide (58). One putative proteolytic cleavage separating fetuin into two chains occurs at site 340 leading to the removal of an Arg residue. The second proteolytic cleavage releasing the connecting peptide (with the phosphate at Ser330) is Leu300. It is therefore tempting to speculate about a possible cross-talk mechanism between Ser330 phosphorylation and fetuin processing. One proposed functional role of this connecting peptide is in an insulin receptor inhibitory function (INSR) (59, 60). The INSR inhibitory activity of matured human fetuin isolated from plasma (assumed without the connecting peptide) is significantly lower compared with recombinant human fetuin (which is a single-chain protein including the connecting peptide). Because it has been reported that 7–20% of circulating fetuin carries phosphorylation at Ser330 (14), it is tempting to speculate that a similar portion of human fetuin circulates as a single-chain protein containing the connecting peptide (14, 57). The existence of such a minor, albeit metabolically more active pool of fetuin could be important in the pathology of type 2 diabetes which relates to acquired or inherited defects in the insulin receptor signaling cascade. Our data suggest a different scenario. We showed in our earlier study and here, that human serum fetuin exists exclusively in the two-chain form architecture, however it still contains the connecting peptide. The proteolytic cleavage separating fetuin into two chains occurs at aforementioned position 340 and completely removes the Arg residue. Therefore, the modification of phosphorylation site Ser330 does not seem to correlate with the absence/presence of the connecting peptide and a putative regulatory function of the phosphate at Ser330 has likely a different mechanism.

##### Sepsis Specific Alterations in the Proteoforms of Human Fetuin

In many diseases, including acute and chronic inflammatory diseases, several acute phase proteins display altered glycosylation (61). Human fetuin has been classified as a negative acute phase glycoprotein, however, whether and how inflammatory processes affect fetuin glycosylation has not been described yet. Here, we observed in fetuin purified from septic patient donors a statistically significant increase of fucosylation on N-glycosylation site Asn176 (B). Notably, two septic patients, M7 and F7, did not show increased fucosylation. Both these patients were considerably younger compared with the other patients (18 and 19 years old). It has been previously hypothesized that N-glycan profiles are affected by age and gender (62–65). Although arguably on a small dataset, we did observe a correlation in our data between the extent of fucosylation and age. However, the number of data points showing the correlation between fucosylation and age was not enough to perform a proper statistical evaluation. Nonetheless, this further supports that the discovery and development of biomarkers should seriously take into consideration factors like gender and age of the donors. Whether the increased fucosylation has a functional consequence or is barely a diagnostic marker of “diseases-state” needs further exploration.

In conclusion, we analyzed here for the first time fetuin samples extracted from twenty individual donors, 10 healthy and 10 suffering from a septic shock. This personalized in-depth proteoform profiling provided a wealth of data on fetuin processing, fetuin phosphorylation and fetuin N- and O-glycosylation. One of the most intriguing findings of our study is that the common fetuin gene polymorphism affects the corresponding proteins' proteoform profiles.

In most genomics and proteomics studies on fetuin so far consequences of the gene polymorphisms, and fetuin processing have been largely ignored. We feel that apparent discrepancies on fetuin reported in the literature, need to be readdressed, taking into account the donor's individual genotype, physiological state, and fetuin processing state. It has been often suggested that fetuin is a protein sensitive to various physiological states, but whether its clinical potential is strong, deserves to be further investigated. Our data clearly demonstrates how a single gene can lead to a very broad range of proteoforms, which all may be functionally differentially active. At the proteoform level there seem to be no boring genes, as even fetuin, characterized already 75 years ago, still exposes new and exciting features when studied in human individuals exhibiting different genotypes and physiological states.

## DATA AVAILABILITY

The mass spectrometry data have been deposited to the ProteomeXchange Consortium via the PRIDE (66) partner repository with the dataset identifier PXD012465 and 10.6019/PXD012465. As PRIDE does not fully support glycopeptide data Byonic results, in the form of freely available Byonic Preview files, containing all identified and assigned spectra are available at the following link: https://figshare.com/s/c3ef7dba6c2079e0402f.

## Supplementary Material

supplemental Table S2

Supplemental Figures and Legends to Supplementary Tables

Annotated MSMS spectra

MS/MS spectra of peptides harboring the glycosylation site T/S256

Excel file containing all supplementary tables

## Footnotes

^1^ The abbreviations used are:

CVD: cardiovascular disease
ABC: ammonium bicarbonate
AMAC: ammonium acetate
ACN: Acetonitrile
Asn: Asparagine
CAT: weak cation exchange
CKD: chronic kidney disease
dHex: deoxyhexose
EMR: Extended mass range Exactive Plus Orbitrap instrument
ELISA: enzyme-linked immunosorbent assays
ESI: electrospray ionization
EThcD: electron-transfer combined with higher-energy collision dissociation
FA: formic acid
GlcNAc: N-acetylglucosamine
HexNAc: N-acetylhexosamine
IAA: iodoacetamide
IEX: ion-exchange chromatography
INSR: insulin receptor
Met: Methionine
MS: mass spectrometry
Neu5Ac: N-acetylneuraminic acid
Pho: phosphorylation
PTMs: post-translational modifications
Ser: Serine
SNPs: single nucleotide polymorphisms
T2DM: type 2 diabetes
Thr: Threonine
XIC: extracted ion chromatogram
WAX: weak anion exchange.
