# Supplementary material for: Glycoproteogenomics: A Frequent Gene Polymorphism Affects the Glycosylation Pattern of the Human Serum Fetuin/α-2-HS-Glycoprotein
Source: Mol Cell Proteomics. 2019 May 16;18(8):1479–90. doi: 10.1074/mcp.RA119.001411 (PMC6683009; doi:10.1074/mcp.RA119.001411)
Supplement: supplemental Table S2 [file RA119.001411_index.html]

Supplement to Glycoproteogenomics: a frequent gene polymorphism affects the glycosylation pattern of the human serum fetuin/α-2-HS-glycoprotein | Molecular & Cellular Proteomics

## Supplemental Data

- Supplemental Figures and Legends to Supplementary Tables - Supplemental Figures and Legends to Supplementary Tables
- Annotated MSMS spectra - Annotated MSMS spectra
- MS/MS spectra of peptides harboring the glycosylation site T/S256 - MS/MS spectra of peptides harboring the glycosylation site T/S256
- Excel file containing all supplementary tables - Excel file containing all supplementary tables
