# Supplementary material for: Glycoproteogenomics: A Frequent Gene Polymorphism Affects the Glycosylation Pattern of the Human Serum Fetuin/α-2-HS-Glycoprotein
Source: Mol Cell Proteomics. 2019 May 16;18(8):1479–90. doi: 10.1074/mcp.RA119.001411 (PMC6683009; doi:10.1074/mcp.RA119.001411)
Supplement: Supplemental Figures and Legends to Supplementary Tables [file 143896_2_supp_328328_prfpsr.pdf]

Glycoproteogenomics: a frequent gene polymorphism affects the glycosylation pattern of the human serum fetuin/α-2-HS-glycoprotein

Yu-Hsien Lin[a,b], Jing Zhu[a,b] , Sander Meijer [a,c], Vojtech Franc [a,b]*, and Albert J.R. Heck[a,b]*

[a] Biomolecular Mass Spectrometry and Proteomics, Bijvoet Center for Biomolecular Research and Utrecht Institute for Pharmaceutical Sciences, Utrecht University, Padualaan 8, 3584 CH Utrecht, The Netherlands

[b] Netherlands Proteomics Center, Padualaan 8, 3584 CH Utrecht, The Netherlands

[c] Department of Molecular and Cellular Hemostasis, Sanquin Research, Amsterdam 1066 CX, the Netherlands

* Authors for Correspondence: Vojtech Franc, v.franc@uu.nl (+31302536149) or Albert Heck, a.j.r.heck@uu.nl (+31302536797)

Molecular & Cellular Proteomics

Supporting Information

Index

Supplemental Table Legends……………………………………………………………S-2

Supplemental Figures…………………………………………………………….S-3-S-6

Supplemental File S1

Supplemental File S2

Supplemental Table Legends

Supplemental Table S1: Relative quantification of the peptides containing the mutations and O-glycosylation site Thr/Ser256, obtained for all 10 healthy individuals

Supplemental Table S2: List of annotated proteoforms of fetuin pooled from human sera of various donors

Supplemental Table S3: List of annotated proteoforms of fetuin derived from sample F1 (AHSG*1)

Supplemental Table S4: List of annotated proteoforms of fetuin derived from sample M3 (AHSG*2)

Supplemental Table S5: List of annotated proteoforms of fetuin derived from sample F3 (AHSG*1/2)

Supplemental Table S6: Relative quantification of the peptides containing the mutations and O-glycosylation site Thr/Ser256, obtained for all 10 septic individuals

Supplemental Table S7: List of the ratio of the fucosylated proteoform and its non-fucosylated proteoform calculated from the intensities extracted from the native spectra

Supplemental Table S8: List of the ratio of the fucosylated and non-fucosylated peptides containing N-glycosylation site N176 calculated from their peak areas

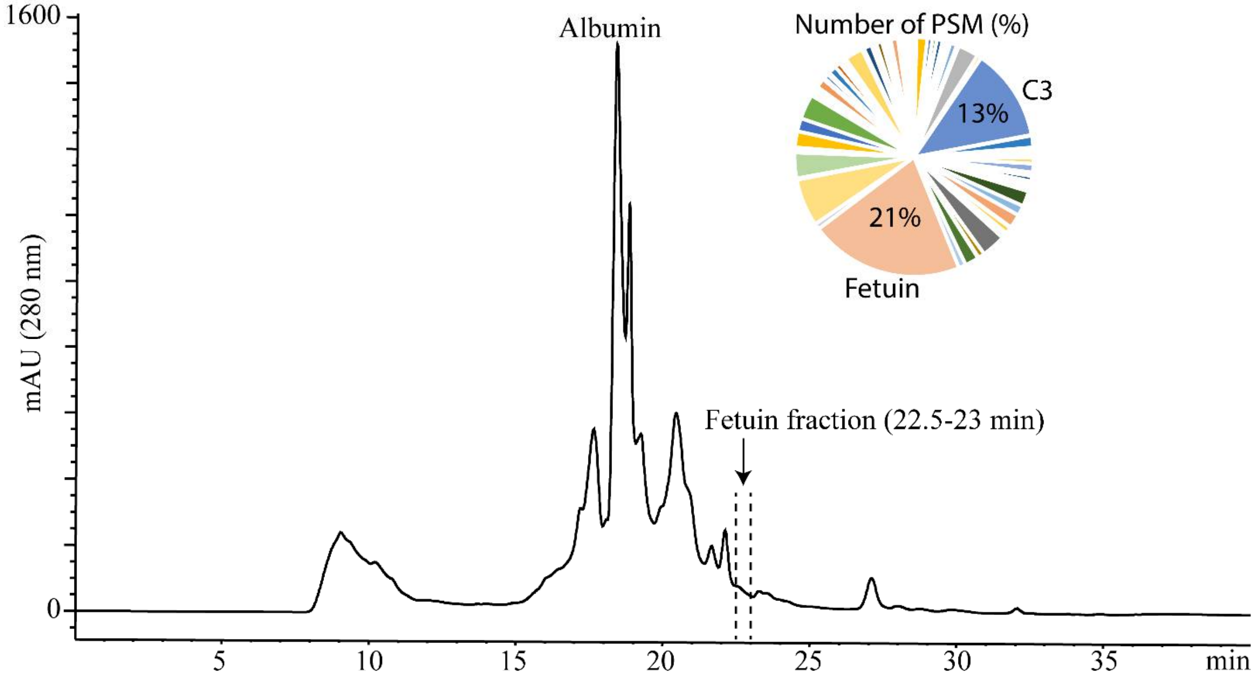

Supplemental Figure S1. Ion exchange based fractionation of human serum from one healthy individual. The fetuin fraction was collected in the time window 22.5-23 min. The proteins in this fractions were identified and quantified by bottom-up proteomics (using number of peptide spectral matches – PSM). The pie chart depicted in the inset reveals that fetuin is not the only, albeit the predominant protein (21%), in this fraction.

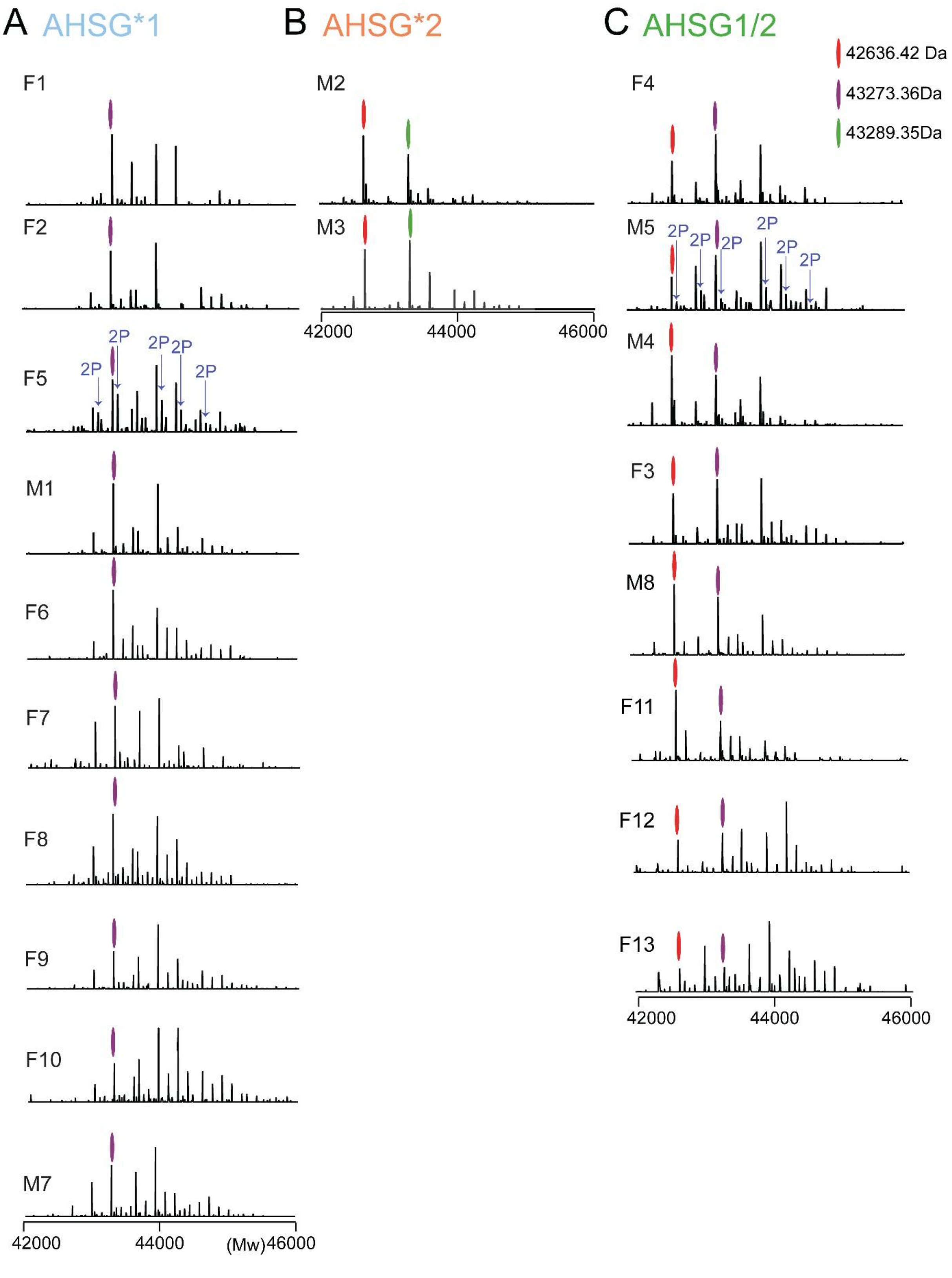
A AHSG*1
B AHSG*2
C AHSG1/2
42636.42 Da
43273.36Da
43289.35Da
F1
F2
F5
M1
F6
F7
F8
F9
F10
M7
M2
M3
F4
M5
M4
F3
M8
F11
F12
F13
2P
42000
44000
46000
(Mw)

Supplemental Figure S2. Different genotypes lead to different proteoform profiles. Overview of all zero-charge deconvoluted native mass spectra of human fetuin derived from the 10 healthy and 10 septic patient donors. The spectra are classified into the three fetuin genotypes A. AHSG*1, B. AHSG*2 and C. AHSG1/2 based on the signature peaks assigned with color codes (mass annotated proteoforms). The proteoforms marked with 2P indicate that the PTM composition contains 2 phosphate moieties.

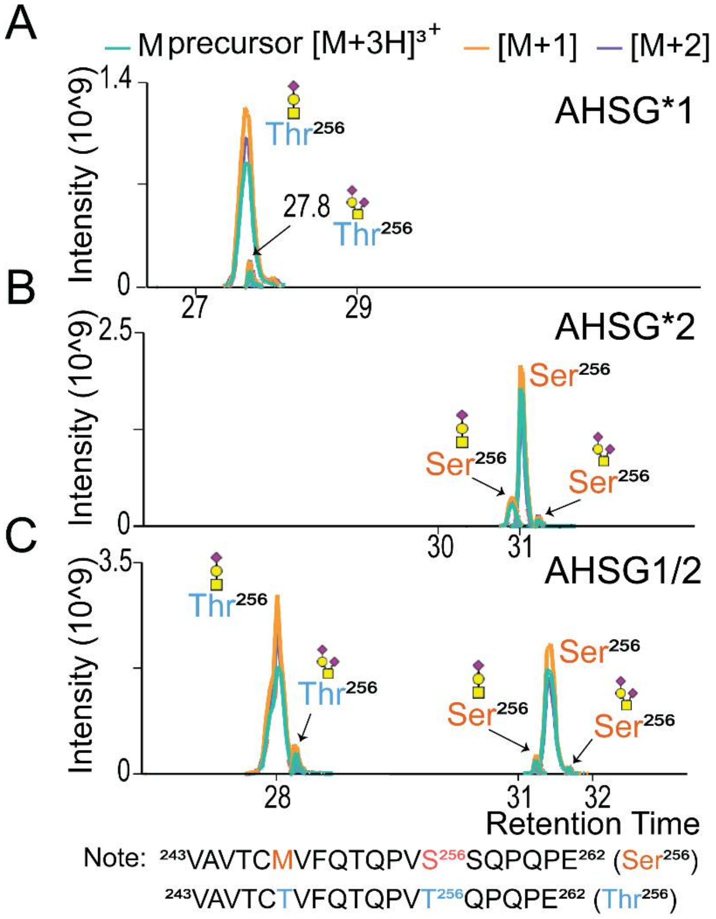

Supplemental Figure S3. Quantification of the peptide signatures containing the mutations and O-glycosylation site Thr/Ser256. Extracted ion chromatograms (XICs) of the peptides containing the O-glycosylation site A. Thr256 on AHSG*1 fetuin, B. Ser256 on AHSG*2 fetuin and C. both Thr256 and Ser256 on AHSG1/2 fetuin.

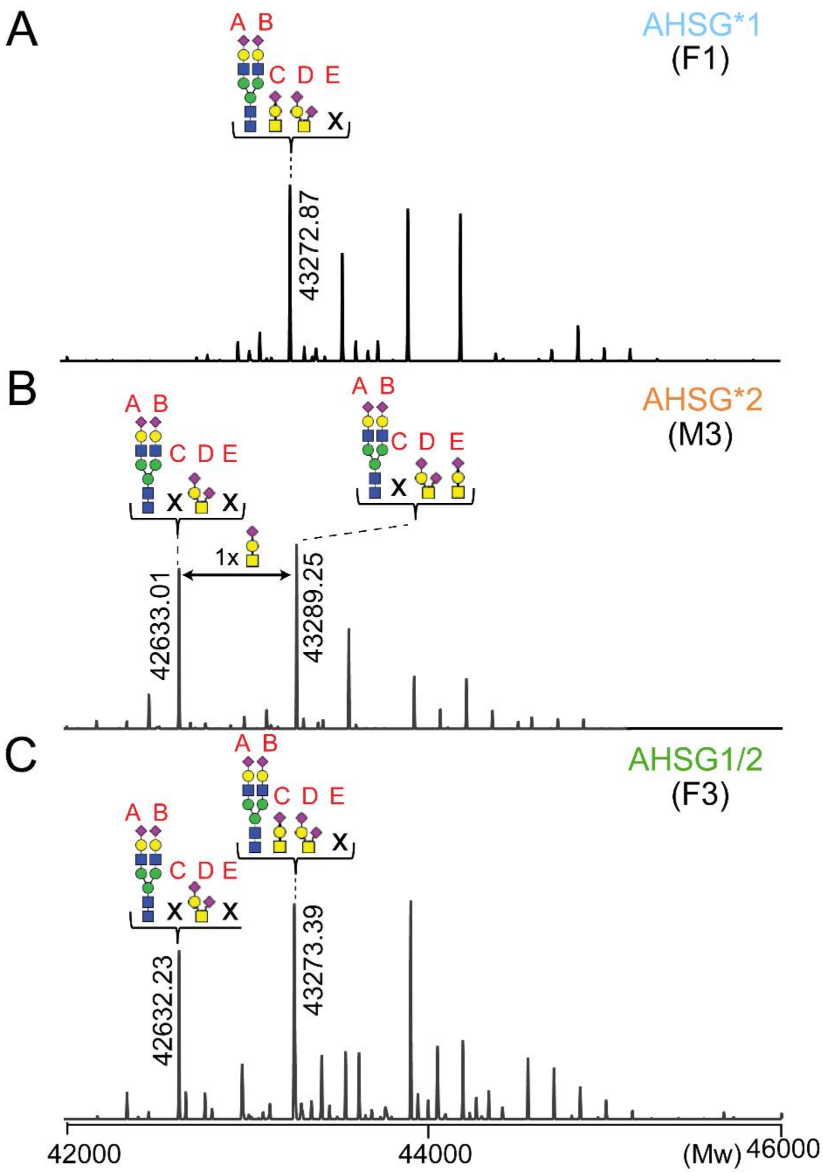

Supplemental Figure S4. Proteoform profiles originating from individuals representative for the fetuin purified from the serum of A. AHSG*1 donor (F1) B. AHSG*2 donor (M3), and C. AHSG1/2 donor (F3). The depicted glycan combination in a site-specific manner shown in A, B and C corresponds to the signature peaks for the three different genotypes. (A=Asn156, B=Asn176, C=Thr256, D=Thr270, and E=Ser346, X means unmodified glycosylation site.)
