# Supplementary material for: Glycoproteogenomics: A Frequent Gene Polymorphism Affects the Glycosylation Pattern of the Human Serum Fetuin/α-2-HS-Glycoprotein
Source: Mol Cell Proteomics. 2019 May 16;18(8):1479–90. doi: 10.1074/mcp.RA119.001411 (PMC6683009; doi:10.1074/mcp.RA119.001411)
Supplement: Annotated MSMS spectra [file 143896_2_supp_328329_prfpsr.pdf]

Human fetuin sample F5 and M5: MS/MS spectra of phosphorylation site S330

MS/MS spectra of phosphorylation site S330

A Sample:F5; Scan:10134; Precursor(m/z): 694.6796; Charge state: 3+

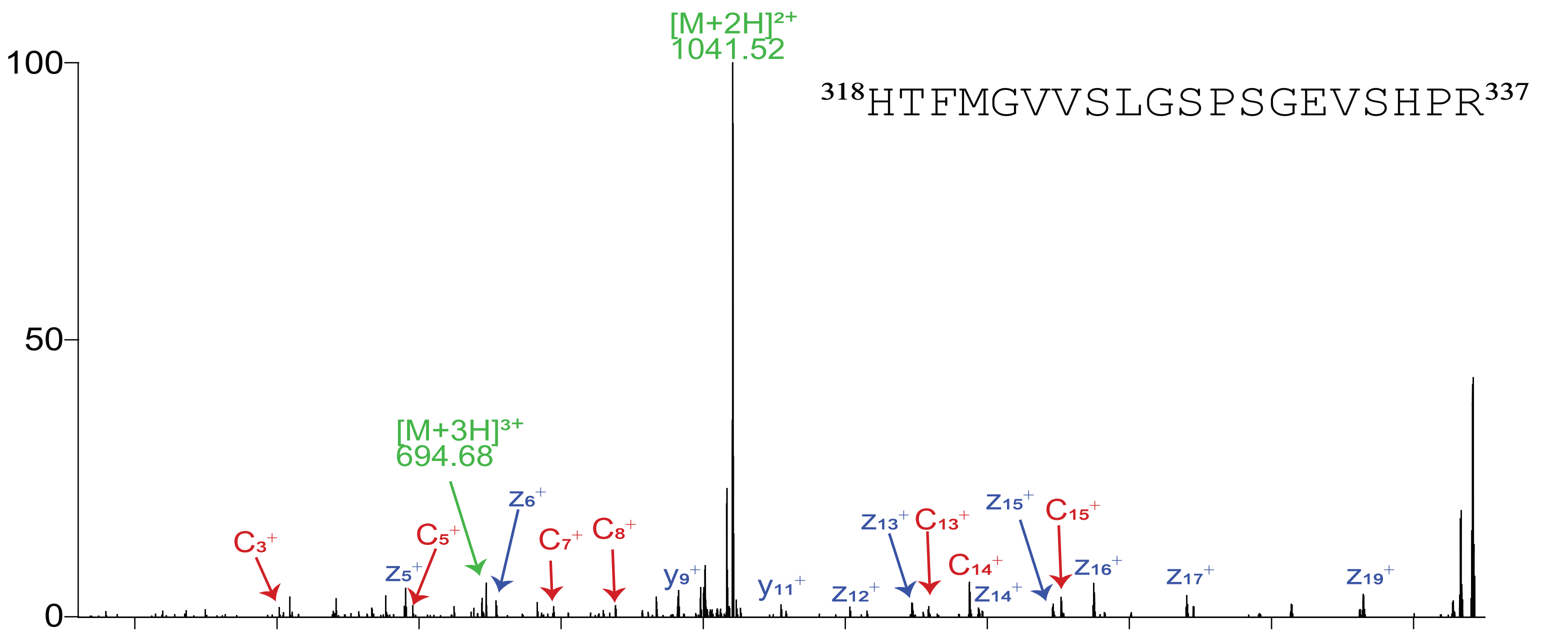

B Sample:F5; Scan:11396; Precursor(m/z): 721.34; Charge state: 3+

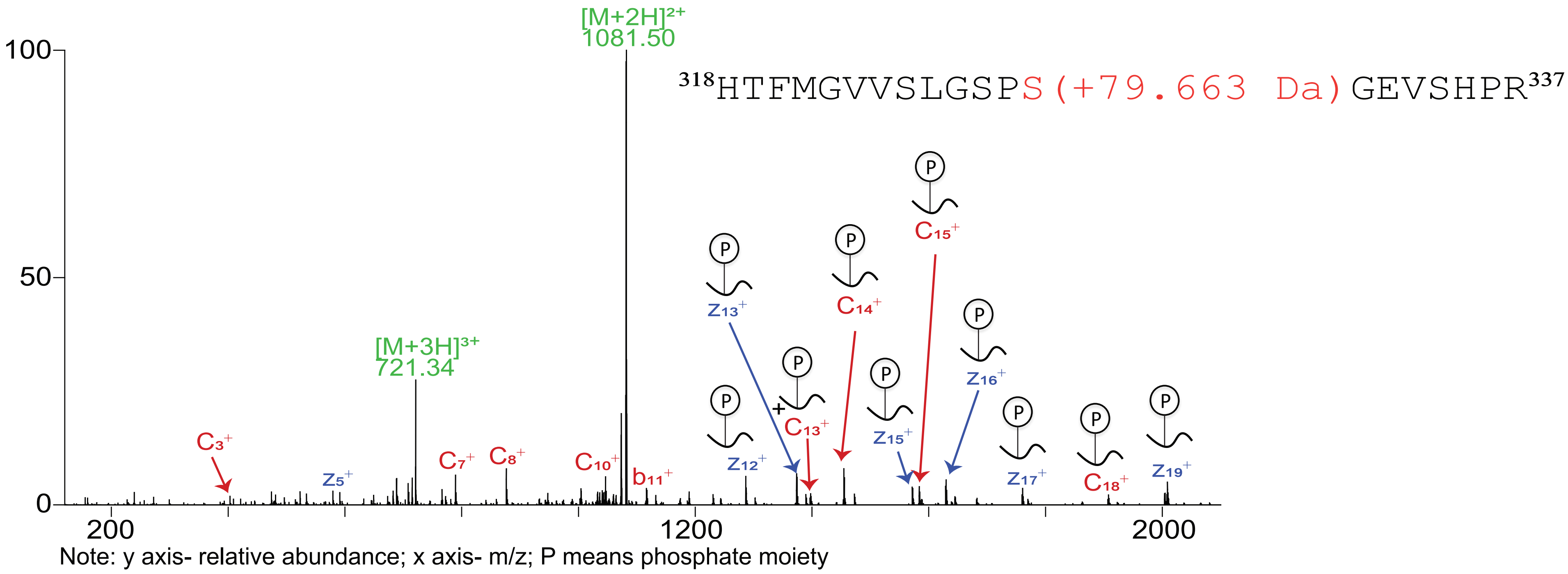

Note: y axis- relative abundance; x axis- m/z; P means phosphate moiety

Fragment ions matches for Figure A

#1	b+	c+	Seq.	y+	z+	#2
1	138.07	155.09	H			20
2	239.11	256.14	T	1943.96	1927.95	19
3	386.18	403.21	F	1842.92	1826.90	18
4	517.22	534.25	M	1695.85	1679.83	17
5	574.24	591.27	G	1564.81	1548.79	16
6	673.31	690.34	V	1507.79	1491.77	15
7	772.38	789.41	V	1408.72	1392.70	14
8	859.41	876.44	S	1309.65	1293.63	13
9	972.50	989.52	L	1222.62	1206.60	12
10	1029.52	1046.55	G	1109.53	1093.51	11
11	1116.55	1133.58	S	1052.51	1036.49	10
12	1213.60	1230.63	P	965.48	949.46	9
13	1300.64	1317.66	S	868.43	852.41	8
14	1357.66	1374.68	G	781.40	765.38	7
15	1486.70	1503.73	E	724.37	708.35	6
16	1585.77	1602.79	V	595.33	579.31	5
17	1672.80	1689.83	S	496.26	480.24	4
18	1809.86	1826.89	H	409.23	393.21	3
19	1906.91	1923.94	P	272.17	256.15	2
20			R	175.12	159.10	1

Fragment ions matches for Figure B

#1	b+	c+	Seq.	y+	z+	#2
1	138.07	155.09	H			20
2	239.11	256.14	T	2023.93	2007.91	19
3	386.18	403.21	F	1922.88	1906.86	18
4	517.22	534.25	M	1775.81	1759.80	17
5	574.24	591.27	G	1644.77	1628.76	16
6	673.31	690.34	V	1587.75	1571.73	15
7	772.38	789.41	V	1488.68	1472.67	14
8	859.41	876.44	S	1389.62	1373.60	13
9	972.50	989.52	L	1302.58	1286.57	12
10	1029.52	1046.55	G	1189.50	1173.48	11
11	1116.55	1133.58	S	1132.48	1116.46	10
12	1213.60	1230.63	P	1045.45	1029.43	9
13	1380.60	1397.63	S-Phospho	948.39	932.37	8
14	1437.62	1454.65	G	781.40	765.38	7
15	1566.67	1583.69	E	724.37	708.35	6
16	1665.73	1682.76	V	595.33	579.31	5
17	1752.77	1769.79	S	496.26	480.24	4
18	1889.83	1906.85	H	409.23	393.21	3
19	1986.88	2003.90	P	272.17	256.15	2
20			R	175.12	159.10	1

MS/MS spectra of phosphorylation site S330

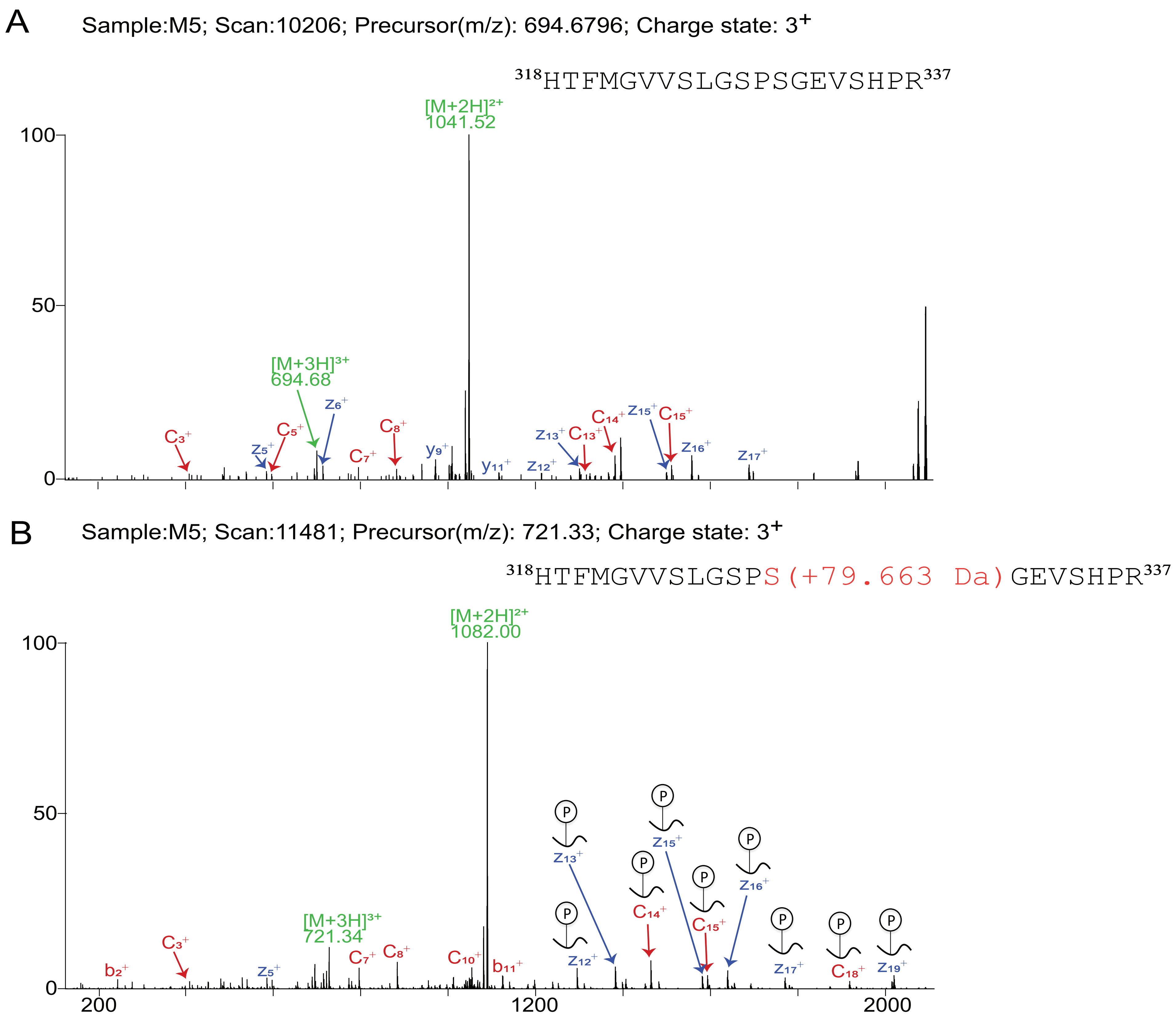

Note: y axis- relative abundance; x axis- m/z; P means phosphate moiety

Fragment ions matches for Figure A

#1	b+	c+	Seq.	y+	z+	#2
1	138.07	155.09	H			20
2	239.11	256.14	T	1943.96	1927.95	19
3	386.18	403.21	F	1842.92	1826.90	18
4	517.22	534.25	M	1695.85	1679.83	17
5	574.24	591.27	G	1564.81	1548.79	16
6	673.31	690.34	V	1507.79	1491.77	15
7	772.38	789.41	V	1408.72	1392.70	14
8	859.41	876.44	S	1309.65	1293.63	13
9	972.50	989.52	L	1222.62	1206.60	12
10	1029.52	1046.55	G	1109.53	1093.51	11
11	1116.55	1133.58	S	1052.51	1036.49	10
12	1213.60	1230.63	P	965.48	949.46	9
13	1300.64	1317.66	S	868.43	852.41	8
14	1357.66	1374.68	G	781.40	765.38	7
15	1486.70	1503.73	E	724.37	708.35	6
16	1585.77	1602.79	V	595.33	579.31	5
17	1672.80	1689.83	S	496.26	480.24	4
18	1809.86	1826.89	H	409.23	393.21	3
19	1906.91	1923.94	P	272.17	256.15	2
20			R	175.12	159.10	1

Fragment ions matches for Figure B

#1	b+	c+	Seq.	y+	z+	#2
1	138.07	155.09	H			20
2	239.11	256.14	T	2023.93	2007.91	19
3	386.18	403.21	F	1922.88	1906.86	18
4	517.22	534.25	M	1775.81	1759.80	17
5	574.24	591.27	G	1644.77	1628.76	16
6	673.31	690.34	V	1587.75	1571.73	15
7	772.38	789.41	V	1488.68	1472.67	14
8	859.41	876.44	S	1389.62	1373.60	13
9	972.50	989.52	L	1302.58	1286.57	12
10	1029.52	1046.55	G	1189.50	1173.48	11
11	1116.55	1133.58	S	1132.48	1116.46	10
12	1213.60	1230.63	P	1045.45	1029.43	9
13	1380.60	1397.63	S-Phospho	948.39	932.37	8
14	1437.62	1454.65	G	781.40	765.38	7
15	1566.67	1583.69	E	724.37	708.35	6
16	1665.73	1682.76	V	595.33	579.31	5
17	1752.77	1769.79	S	496.26	480.24	4
18	1889.83	1906.85	H	409.23	393.21	3
19	1986.88	2003.90	P	272.17	256.15	2
20			R	175.12	159.10	1
