# Supplementary material for: Glycoproteogenomics: A Frequent Gene Polymorphism Affects the Glycosylation Pattern of the Human Serum Fetuin/α-2-HS-Glycoprotein
Source: Mol Cell Proteomics. 2019 May 16;18(8):1479–90. doi: 10.1074/mcp.RA119.001411 (PMC6683009; doi:10.1074/mcp.RA119.001411)
Supplement: MS/MS spectra of peptides harboring the glycosylation site T/S256 [file 143896_2_supp_328330_prfpsr.pdf]

Human fetuin sample: MS/MS spectra of glycosylation site T/S256

MS/MS spectra of glycosylation site T256

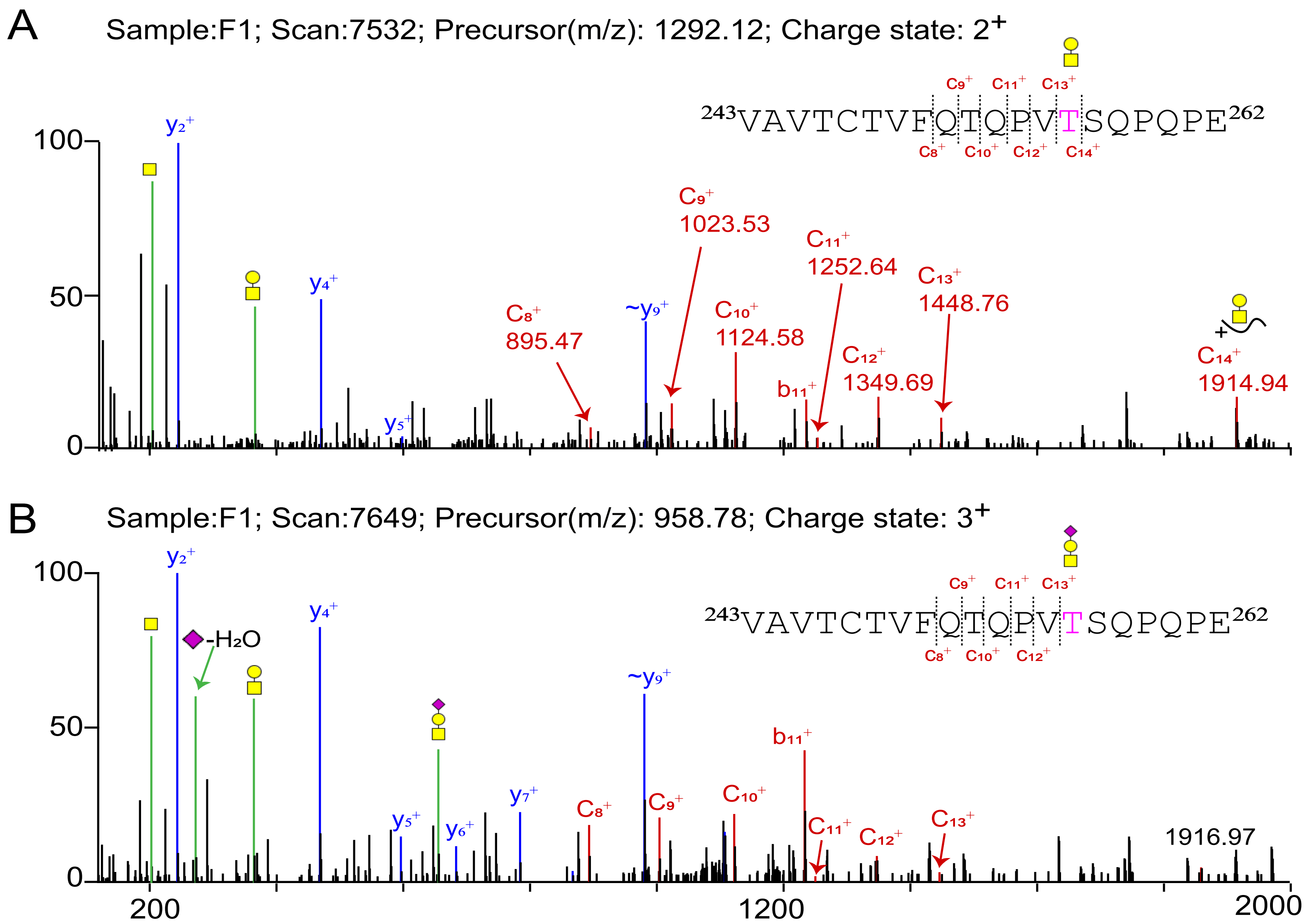

Note: y axis- relative abundance; x axis- m/z; ~ means gase-phase loss of O-glycan

Fragment ions matches for Figure A

#1	b+	c+	Seq.	y+	z+	#2
1	100.08	117.10	V			20
2	171.11	188.14	A	2483.15	2467.13	19
3	270.18	287.21	V	2412.11	2396.09	18
4	371.23	388.26	T	2313.04	2297.03	17
5	531.26	548.29	C-Carbamidomethyl	2212.00	2195.98	16
6	632.31	649.33	T	2051.97	2035.95	15
7	731.38	748.40	V	1950.92	1934.90	14
8	878.44	895.47	F	1851.85	1835.83	13
9	1006.50	1023.53	Q	1704.78	1688.76	12
10	1107.55	1124.58	T	1576.72	1560.70	11
11	1235.61	1252.64	Q	1475.67	1459.66	10
12	1332.66	1349.69	P	1347.62	1331.60	9
13	1431.73	1448.76	V	1250.56	1234.54	8
14	1897.91	1914.94	T-HexNAc(1)Hex(1)	1151.50	1135.48	7
15	1984.94	2001.97	S	685.32	669.30	6
16	2113.00	2130.03	Q	598.28	582.26	5
17	2210.05	2227.08	P	470.22	454.21	4
18	2338.11	2355.14	Q	373.17	357.15	3
19	2435.16	2452.19	P	245.11	229.09	2
20			E	148.06	132.04	1

Fragment ions matches for Figure B

#1	b+	c+	Seq.	y+	z+	#2
1	100.08	117.10	V			20
2	171.11	188.14	A	2774.24	2758.23	19
3	270.18	287.21	V	2703.21	2687.19	18
4	371.23	388.26	T	2604.14	2588.12	17
5	531.26	548.29	C-Carbamidomethyl	2503.09	2487.07	16
6	632.31	649.33	T	2343.06	2327.04	15
7	731.38	748.40	V	2242.01	2225.99	14
8	878.44	895.47	F	2142.94	2126.93	13
9	1006.50	1023.53	Q	1995.88	1979.86	12
10	1107.55	1124.58	T	1867.82	1851.80	11
11	1235.61	1252.64	Q	1766.77	1750.75	10
12	1332.66	1349.69	P	1638.71	1622.69	9
13	1431.73	1448.76	V	1541.66	1525.64	8
14	2189.01	2206.03	T-HexNAc(1)Hex(1)NeuAc(1)	1442.59	1426.57	7
15	2276.04	2293.06	S	685.32	669.30	6
16	2404.10	2421.12	Q	598.28	582.26	5
17	2501.15	2518.18	P	470.22	454.21	4
18	2629.21	2646.23	Q	373.17	357.15	3
19	2726.26	2743.29	P	245.11	229.09	2
20			E	148.06	132.04	1

MS/MS spectra of glycosylation site S256

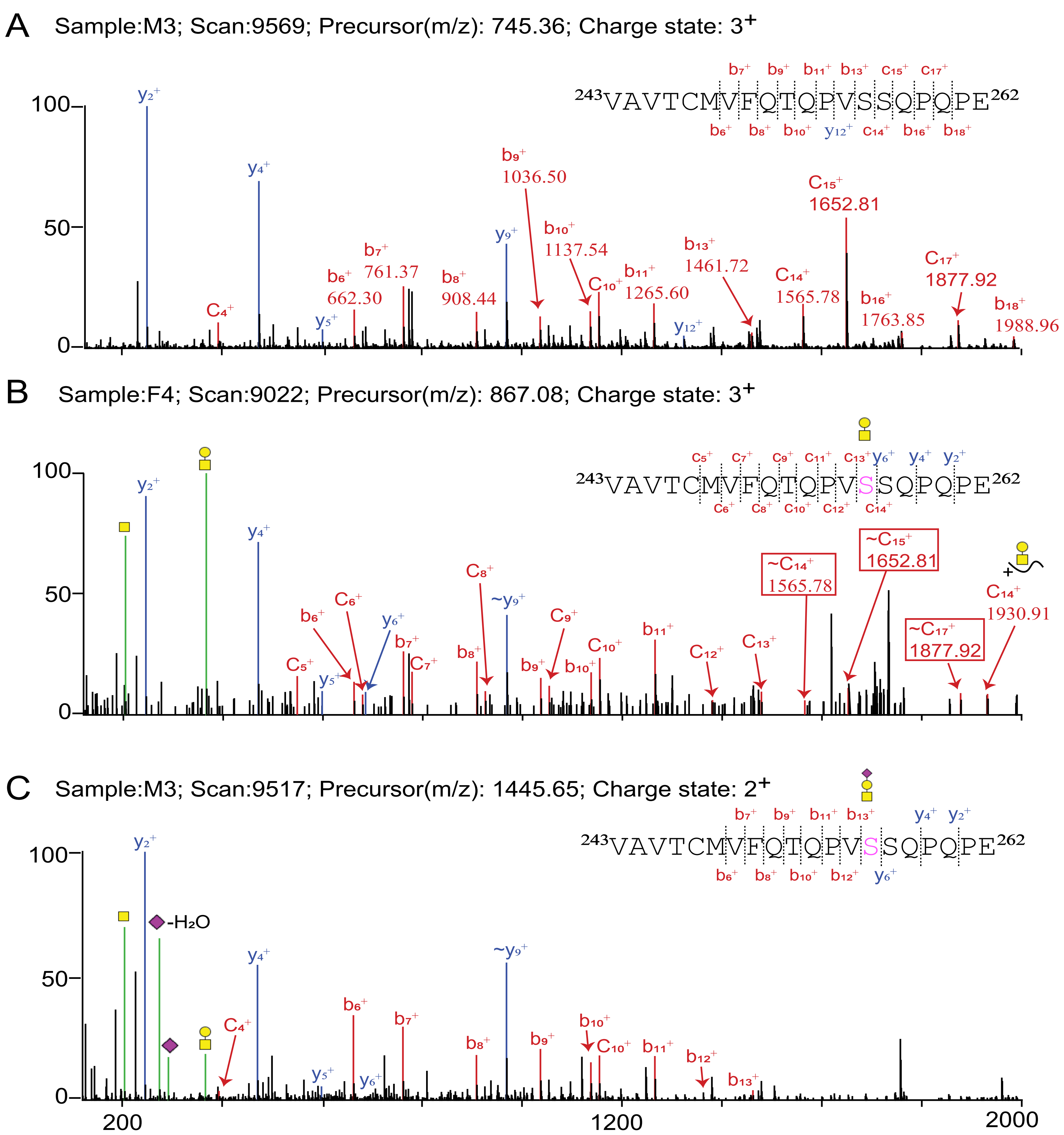

Note: y axis- relative abundance; x axis- m/z; ~ means gase-phase loss of O-glycan

Fragment ions matches for Figure A

#1	b+	c+	Seq.	y+	z+	#2
1	100.08	117.10	V			20
2	171.11	188.14	A	2133.99	2117.98	19
3	270.18	287.21	V	2062.96	2046.94	18
4	371.23	388.26	T	1963.89	1947.87	17
5	531.26	548.29	C-Carbamidomethyl	1862.84	1846.82	16
6	662.30	679.33	M	1702.81	1686.79	15
7	761.37	778.39	V	1571.77	1555.75	14
8	908.44	925.46	F	1472.70	1456.68	13
9	1036.50	1053.52	Q	1325.63	1309.61	12
10	1137.54	1154.57	T	1197.57	1181.56	11
11	1265.60	1282.63	Q	1096.53	1080.51	10
12	1362.65	1379.68	P	968.47	952.45	9
13	1461.72	1478.75	V	871.42	855.40	8
14	1548.75	1565.78	S	772.35	756.33	7
15	1635.79	1652.81	S	685.32	669.30	6
16	1763.85	1780.87	Q	598.28	582.26	5
17	1860.90	1877.92	P	470.22	454.21	4
18	1988.96	2005.98	Q	373.17	357.15	3
19	2086.01	2103.04	P	245.11	229.09	2
20			E	148.06	132.04	1

Fragment ions matches for Figure B

#1	b+	c+	Seq.	y+	z+	#2
1	100.08	117.10	V			20
2	171.11	188.14	A	2499.13	2483.11	19
3	270.18	287.21	V	2428.09	2412.07	18
4	371.23	388.26	T	2329.02	2313.00	17
5	531.26	548.29	C-Carbamidomethyl	2227.97	2211.95	16
6	662.30	679.33	M	2067.94	2051.92	15
7	761.37	778.39	V	1936.90	1920.88	14
8	908.44	925.46	F	1837.83	1821.82	13
9	1036.50	1053.52	Q	1690.77	1674.75	12
10	1137.54	1154.57	T	1562.71	1546.69	11
11	1265.60	1282.63	Q	1461.66	1445.64	10
12	1362.65	1379.68	P	1333.60	1317.58	9
13	1461.72	1478.75	V	1236.55	1220.53	8
14	1913.89	1930.91	S-HexNAc(1)Hex(1)	1137.48	1121.46	7
15	2000.92	2017.95	S	685.32	669.30	6
16	2128.98	2146.00	Q	598.28	582.26	5
17	2226.03	2243.06	P	470.22	454.21	4
18	2354.09	2371.12	Q	373.17	357.15	3
19	2451.14	2468.17	P	245.11	229.09	2
20			E	148.06	132.04	1

Fragment ions matches for Figure C

#1	b+	c+	Seq.	y+	z+	#2
1	100.08	117.10	V			20
2	171.11	188.14	A	2790.22	2774.20	19
3	270.18	287.21	V	2719.18	2703.17	18
4	371.23	388.26	T	2620.12	2604.10	17
5	531.26	548.29	C-Carbamidomethyl	2519.07	2503.05	16
6	662.30	679.33	M	2359.04	2343.02	15
7	761.37	778.39	V	2228.00	2211.98	14
8	908.44	925.46	F	2128.93	2112.91	13
9	1036.50	1053.52	Q	1981.86	1965.84	12
10	1137.54	1154.57	T	1853.80	1837.78	11
11	1265.60	1282.63	Q	1752.75	1736.74	10
12	1362.65	1379.68	P	1624.70	1608.68	9
13	1461.72	1478.75	V	1527.64	1511.62	8
14	2204.98	2222.01	S-HexNAc(1)Hex(1)NeuAc(1)	1428.57	1412.56	7
15	2292.01	2309.04	S	685.32	669.30	6
16	2420.07	2437.10	Q	598.28	582.26	5
17	2517.13	2534.15	P	470.22	454.21	4
18	2645.18	2662.21	Q	373.17	357.15	3
19	2742.24	2759.26	P	245.11	229.09	2
20			E	148.06	132.04	1
